# Complete genome sequence of *Trueperella pyogenes* strain Arash114, isolated from the uterus of a water buffalo (*Bubalus bubalis*) in Iran

**DOI:** 10.1186/s13104-021-05734-1

**Published:** 2021-08-23

**Authors:** Iradj Ashrafi Tamai, Abdolmajid Mohammadzadeh, Arash Ghalyanchi Langeroudi, Pezhman Mahmoodi, Zahra Ziafati Kafi, Babak Pakbin, Taghi Zahraei Salehi

**Affiliations:** 1 Department of Pathobiology, Faculty of Veterinary Science, Bu-Ali Sina University, Hamedan, Iran; 2 Department of Microbiology and Immunology, Faculty of Veterinary Medicine, University of Tehran, Tehran, Iran; 3 Medical Microbiology Research Center, Qazvin University of Medical Sciences, Qazvin, Iran

**Keywords:** *Trueperella pyogenes*, Complete genome sequencing, Uterus infection, Water buffalo

## Abstract

**Objective:**

*Trueperella pyogenes* has been considered a major causative agent of metritis, abortion, and death in a broad range of domestic and wild animals, including cattle, swine, sheep, goats, camels, buffalo, deer, antelopes, reptiles, and birds.

**Data description:**

Here, we report the complete chromosome sequence of *Trueperella pyogenes* strain Arash114, isolated from the uterus of a water buffalo (*Bubalus bubalis*) died due to the infection caused by this pathogen. The genome assembly comprised 2,338,282 bp, with a 59.5% GC content. Annotation of the genome showed 46 tRNA genes, 6 rRNA, 1 CRISPR and 2059 coding sequences. Also, several genes coding for antimicrobial resistance such as *tetW* and virulence factor including *plo*, *nanH*, *nanP*, *cbp* and 4 fimbrial proteins were found. This study will advance our knowledge regarding the metabolism, virulence factors, antibiotic resistance and evolution of Arash114 strain and serve as an appropriate template for future researches.

## Objective

*Trueperella pyogenes*, formerly known as *Actinomyces pyogenes* and *Arcanobacterium pyogenes*, has recently been reclassified based on distinctive *16S rRNA* gene sequences. This bacterium is an irregular, nonmotile, non-spore-forming, aerobic, commensal, Gram-positive, short, rod-shaped bacterium which is normally isolated from the upper respiratory, urogenital, and gastrointestinal tracts [1–3]. *T. pyogenes* is proposed as a globally distributed secondary pathogen which may cause diseases such as acute and summer mastitis, metritis, clinical and subclinical endometritis, cutaneous and visceral abscesses, arthritis, pneumonia, endocarditis, osteomyelitis, and several other suppurative infectious diseases in a broad range of domestic and wild animals, including cattle, swine, sheep, goats, camels, buffalo, deer, antelopes, reptiles, and birds. Severe infections caused by *T. pyogenes* have rarely been reported and are usually associated with occupational exposure, due to the bacterium is not the part of the human normal flora bacteria [4–8].

Several pathogenic properties are known in *T. pyogenes* which can increase its pathogenicity. Pyolysin (*plo*), as one of its major virulence factors, causes hemolysis and cytolysis of leukocytes. On the other hand, bacterial adhesion to epithelial cells and colonization, as well as degradation of DNA and sialic acid, are attributed to H and Pneuraminidases (*nanH* and *nanP*) of this bacterium. Different types of fimbriae are expressed by *T. pyogenes*, including A, G, E, and C. These types of fimbriae are required for adherence to membranes and epithelial cells. Collagen-binding proteins (*Cbp*) and fibronectin-binding proteins (*Fbp*) are essential for adhesion to collagen-rich tissues (types 1, 2, and 4) and fibronectins. In addition, the protease and DNase of *T. pyogenes* provide nutrients for the bacteria through degradation of proteins and nucleic acids [1, 9, 10]. Despite this bacterium has been known as an opportunistic pathogen for human and animals for many decades, characterization of *T. pyogenes* genomes remains still necessary.

## Data description

Here, we report the complete chromosome sequence of *T. pyogenes* strain Arash114. The strain was isolated from the uterus of a water buffalo (*Bubalus bubalis*) and is a major causative agent of metritis, abortion, and death. Therefore, we performed whole-genome sequencing. Genomic DNA was extracted using a commercial DNA extraction kit for Gram-positive bacteria according to the manufacturer’s instructions (Bioneer, South Korea). The quantity and quality properties of DNA was measured using Thermo-Fisher Nano-Drop Spectrophotometer model ND1000 (Thermo Fisher Scientific, DE). Sequencing was performed with the Illumina MiSeq platform using paired-end (PE) reads and Nextera library preparation. The sequences were de novo assembled using the CLC Genomics Workbench software (version 8) (Data set 1 and 2) [11, 12]. Genome annotation of the strain was performed using RAST annotation server [13]. Prediction of clustered regularly interspaced short palindromic repeats (CRISPRs) sequences was performed using CRISPRfinder online program [14, 15]. Antimicrobial resistance genes were identified using “ResFinder”, “card” and “NCBI AMRFinderPlus” databases and ABRicate software version 0.8 (https://github.com/tseemann/abricate) [16].

The complete genome of *T. pyogenes* was 2,338,282 bp long, with a GC content of 59.5%. The genome of the isolate was composed of a total of 2137 genes, including 2082 coding sequences (CDs) (total) and 2059 coding genes; 23 are pseudogenes, 6 are rRNAs, 46 are tRNAs, 3 are noncoding RNAs (ncRNAs) (Data file 2) [17], 1 is a clustered regularly interspaced short palindromic repeat (CRISPR) (Data file 3) [18] including 33 spacers (Data file 4) [19], and 16 are frameshifted genes. The genome encodes several known and putative virulence factors, including pyolysin, collagen-binding protein, neuraminidases (*nanH* and *nanP*), and 4 fimbrial proteins (Data file 2) [17]. Different antibiotics have often been used for treatment of *T. pyogenes* infections in veterinary practices [6]. In this study, a tetracycline resistance encoding gene (*tetW*) was detected in the genome of *T. pyogenes* strain Arash114 (Data file 5) [20]. Although, resistance to β-lactams, chloramphenicol and macrolides antibiotics have been reported among the *T. pyogenes* strains [21, 22]. No other specific antibiotic resistance genes were identified in strain Arash114 (Data file 6 and Data file 7) [23, 24]. Machado and Bicalho (2014) reported the complete genome sequence of *T. pyogenes* as an important opportunistic pathogen from livestock. They found several virulence factors such as collagen adhesion, fimbrial proteins, pyolysin and cytotoxin in the isolate [25]. Zhang et al. [26] also isolated and sequenced the complete genome of *T. pyogenes* from livestock and they detected different virulence factor encoding genes including pyolysin, *cbpA*, *fimC*, *nanH*, *nanP* and *fimE* genes. However, this is the first study reported the complete genome sequence of *T. pyogenes* isolated from Water Buffalo (*Bubalus bubalis*) (Table 1).

**Table 1:** Overview of data files

Label	Name of data file/data set	File types (file extension)	Data repository and identifier (DOI or accession number)
Data set 1	Complete genome file of *T. pyogenes* Arash114 strain	Fasta file (.fasta)	ENA/EMBL (https://identifiers.org/insdc.gca:GCA_003055835.1) [11]
Data set 2	Raw sequencing data of *T. pyogenes* Arash114 strain	Fastq file (.fastq.gz)	ENA/EMBL (https://identifiers.org/ncbi/insdc:CP028833) [12]
Data file 2	Gene annotation results of *T. pyogenes* Arash114 strain	Tab separated values (.tsv)	HARVARD Dataverse (10.7910/DVN/HAJYNP) [17]
Data file 3	CRISPR spacers of *T. pyogenes* Arash114 strain	Text file (.txt)	HARVARD Dataverse (10.7910/DVN/HAJYNP) [18]
Data file 4	CRISPR sequences of *T. pyogenes* Arash114 strain	Text file (.txt)	HARVARD Dataverse (10.7910/DVN/HAJYNP) [19]
Data file 5	Antimicrobial resistance genes of *T. pyogenes* Arash114 strain based on ResFinder database	Comma-separated values (.csv)	HARVARD Dataverse (10.7910/DVN/HAJYNP) [20]
Data file 6	Antimicrobial resistance genes of *T. pyogenes* Arash114 strain based on card database	Comma-separated values (.csv)	HARVARD Dataverse (10.7910/DVN/HAJYNP) [23]
Data file 7	Antimicrobial resistance genes of *T. pyogenes* Arash114 strain based on NCBIAMRFinderPlus database	Comma-separated values (.csv)	HARVARD Dataverse (10.7910/DVN/HAJYNP) [24]

The whole genome sequence presented in this study serve as a platform for detection of new genes that may contribute to antibiotic resistance and pathogenicity of *T. pyogenes* strain Arah114. This will advance our knowledge regarding the metabolism, virulence factors, antibiotic resistance and evolution of Arash114 strain and serve as an appropriate template for future researches.

## Limitations

Annotations and genomic analysis of *T. pyogenes* strain Arash114 were performed with validated, novel and robust online and offline bioinformatics tools; consequently, the authors are currently unaware of any drawback and limitations of the data.

## Data Availability

All data files 2–7 described in this data note can be openly and freely accessed on Harvard Dataverse (https://dataverse.harvard.edu) [17–20, 23, 24]. Data sets 1 and 2 can be openly and freely accessed on the ENA/EMBL database. Sequence reads have been deposited in the ENA/EMBL sequence read archive under accession number CP028833 (https://identifiers.org/insdc.gca:GCA_003055835.1) (Data set 2) [12]. The complete genome of *T. pyogenes* Arash114 has been deposited in GenBank under accession number CP028833 (https://identifiers.org/insdc.gca:GCA_003055835.1) (Data set 1) [11]. The Project accession number for the genome sequencing project of *T. pyogenes* strain Arash114 is PRJNA449465. See the Table 1 and references for more details and links to all data.

## Abbreviations

Plo: Pyolysin
Cbp: Collagen-binding proteins
Fbp: Fibronectin-binding proteins
DNA: Deoxyribose nucleic acid
RAST: Rapid Annotations using Subsystems Technology
CRISPRs: Clustered Regularly Interspaced Short Palindromic Repeats
CDs: Coding sequences
